# Systemic nutritional status and its dynamic changes as predictors of response to neoadjuvant immunotherapy in locally advanced MSS/pMMR colorectal cancer

**DOI:** 10.3389/fmed.2026.1803929

**Published:** 2026-03-27

**Authors:** Haining Chen, Zhiyuan Xia, Yefu Shen, Xihua Xie, Ming Qiu, Minghui Lin, Shen Pang, Dongchao Liu, Wei Huang, Sen Zhang

**Affiliations:** 1 Department of Colorectal and Anal Surgery, The First Affiliated Hospital of Guangxi Medical University, Nanning, China; 2 Guangxi Key Laboratory of Enhanced Recovery After Surgery for Gastrointestinal Cancer, Nanning, China

**Keywords:** GNRI, locally advanced colorectal cancer, MSS/pMMR, neoadjuvant immunotherapy, systemic nutritional status

## Abstract

**Background:**

Patients with locally advanced microsatellite-stable/mismatch repair–proficient (MSS/pMMR) colorectal cancer show heterogeneous responses to neoadjuvant immunotherapy, and effective predictive biomarkers are lacking. Systemic nutritional status correlates with host immune function, but its predictive value for immunotherapy response in this population is unclear. This study aimed to explore the value of systemic nutritional indicators and their dynamic changes in predicting complete response (pCR) after neoadjuvant immunotherapy in these patients.

**Methods:**

Clinical data from 255 patients with locally advanced MSS/pMMR colorectal cancer who received neoadjuvant immunotherapy were retrospectively analyzed. Patients were randomly divided into a training cohort and a validation cohort at a ratio of 7:3. In the training cohort, univariate analysis was first performed to screen potential predictive variables, followed by multivariate logistic regression analysis to identify independent predictors of pCR and to construct a predictive model. The discriminative ability, calibration, and clinical utility of the model were evaluated using the area under the receiver operating characteristic curve (AUC), calibration curves, and decision curve analysis. A nomogram was subsequently developed for visualized individualized prediction.

**Results:**

Multivariate analysis identified younger age (OR = 0.96, 95% CI: 0.93–0.99), lower post–neoadjuvant treatment carcinoembryonic antigen (CEA) levels (OR = 0.19, 95% CI: 0.05–0.70), and an increase in the Geriatric Nutritional Risk Index (GNRI) during treatment (OR = 1.07, 95% CI: 1.02–1.12) as independent predictors of achieving pCR. The logistic regression model incorporating these three variables demonstrated good predictive performance, with AUCs of 0.76 in the training cohort and 0.80 in the validation cohort. The model showed good calibration, and decision curve analysis indicated favorable net clinical benefit. The resulting nomogram provided a practical reference tool for individualized prediction of pCR probability.

**Conclusion:**

Age, post–neoadjuvant treatment CEA levels, and changes in the GNRI were independent predictors of pathological complete response following neoadjuvant immunotherapy in patients with locally advanced MSS/pMMR colorectal cancer. The predictive model and nomogram presented in this study provide a reference for clinical practice and provide a novel perspective for future studies combining nutritional interventions with immunotherapy.

## 1 Introduction

Colorectal cancer (CRC) is one of the most common malignancies of the digestive system, ranking third in incidence and fourth in cancer-related mortality worldwide (1). Currently, neoadjuvant therapy followed by surgical resection has become the standard treatment strategy for patients with locally advanced rectal cancer (2). For patients with microsatellite instability–high (MSI-H) or mismatch repair–deficient (dMMR) CRC, treatment with immune checkpoint inhibitors (ICIs) has reached a broad consensus in the oncology community (3–5). In 2025, the Chinese Society of Clinical Oncology (CSCO) further recommended the use of ICIs during the neoadjuvant treatment phase for patients with microsatellite-stable (MSS) or mismatch repair–proficient (pMMR) locally advanced rectal cancer (6).

However, CRC exhibits marked biological heterogeneity, and patients show substantial variability in their responses to neoadjuvant immunotherapy. At present, sensitive and specific predictive biomarkers are still lacking in clinical practice to accurately assess the therapeutic efficacy of neoadjuvant immunotherapy in patients with CRC.

A growing body of evidence has demonstrated that systemic nutritional indicators can serve as effective predictors of response to neoadjuvant chemoradiotherapy in various malignancies (7–10). Adequate systemic nutritional status is a critical reflection of a well-functioning immune system. Immune checkpoint inhibitors exert their antitumor effects by relieving tumor-induced immune suppression and reactivating endogenous antitumor immune responses (11, 12). The Geriatric Nutritional Risk Index (GNRI) is a well-established indicator of systemic nutritional status that objectively and comprehensively reflects overall nutritional reserves and metabolic balance (13, 14). In other gastrointestinal malignancies, such as esophageal and gastric cancer, previous studies have explored the use of GNRI and other systemic nutritional indices to predict the efficacy of neoadjuvant immunotherapy (8, 10, 15–18). However, to date, no studies have specifically focused on the scenario of neoadjuvant immunotherapy for colorectal cancer. Moreover, most existing studies are based on cross-sectional designs and fail to account for the impact of dynamic changes in systemic nutritional status on the predictive performance for neoadjuvant treatment outcomes.

Therefore, the present study aimed to investigate the potential value of systemic nutritional indicators and their dynamic changes in predicting the efficacy of neoadjuvant immunotherapy in patients with locally advanced MSS/pMMR colorectal cancer.

## 2 Materials and methods

### 2.1 Study population

This study was approved by the Institutional Review Board of The First Affiliated Hospital of Guangxi Medical University (Registration number: No. 2026-E0068) and was conducted in accordance with the ethical principles of the Declaration of Helsinki (1964). The requirement for informed consent was waived due to the retrospective nature of the study.

We retrospectively reviewed the clinical data of patients with colorectal malignancies who received neoadjuvant immunotherapy at The First Affiliated Hospital of Guangxi Medical University between November 2022 and December 2025.

The inclusion criteria were as follows: (1) patients with locally advanced colorectal cancer, pathologically confirmed as colorectal adenocarcinoma, who had not received any antitumor treatment prior to their initial visit; (2) patients who received at least one cycle of programed death-1 (PD-1) inhibitor–based immunotherapy after initial diagnosis; (3) tumor molecular classification confirmed as pMMR or MSS by immunohistochemistry, polymerase chain reaction (PCR), or next-generation sequencing (NGS); (4) complete pretreatment imaging studies with clinical staging of T3-T4 and any N stage, and no evidence of distant metastasis; (5) no history of other malignant tumors; (6) patients who subsequently underwent surgical resection after neoadjuvant treatment. The exclusion criteria were as follows: (1) inconsistent mismatch repair (MMR) or microsatellite instability (MSI) status as determined by immunohistochemistry, PCR, or NGS; (2) multiple primary colorectal cancers; (3) incomplete clinical data.

**FIGURE 1:**
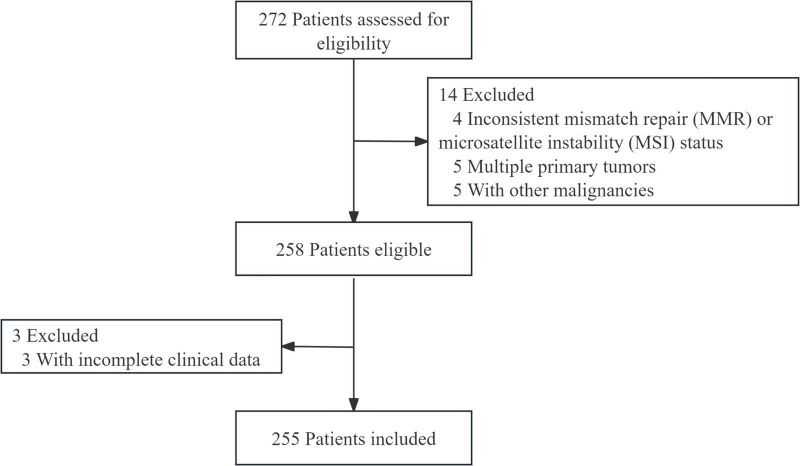
Patient screening flow diagram.

A total of 255 patients with rectal cancer were ultimately included in this study and were randomly divided into a training cohort and a validation cohort at a ratio of 7:3. The patient selection process is illustrated in Figure 1.

### 2.2 Treatment protocol

Patients received neoadjuvant therapy consisting of the CAPOX (capecitabine and oxaliplatin) regimen plus tislelizumab, followed by complete mesocolic excision (CME) or total mesorectal excision (TME) surgery. The CAPOX regimen consisted of intravenous oxaliplatin (130 mg/m^2^) administered on day 1 and oral capecitabine (1,000 mg/m^2^) twice daily on days 1–14 of each 3-week cycle. Tislelizumab (200 mg) is recommended to be administered intravenously on day 1 of each cycle. However, tislelizumab may be skipped in some cycles due to adverse events or patient-related reasons. Details of the information of medication cycles were provided, and statistical analysis revealed no significant difference in treatment cycles between the pCR and non-pCR groups (Supplementary Tables S1, S2).

### 2.3 Clinical outcomes

The primary outcome of this study was whether patients achieved complete response after neoadjuvant immunotherapy. Postoperative pathological specimens were independently reviewed by two experienced pathologists, and tumor regression grade (TRG) was assessed separately by each reviewer according to the 8th edition of the AJCC TRG system (19). Tumor regression was classified as follows: TRG 0: no residual tumor cells (pathological complete response, pCR); TRG 1: single tumor cells or small clusters of residual cancer cells (non-pCR); TRG 2: residual tumor cells present (non-pCR); TRG 3: minimal or no tumor cell destruction (non-pCR). The final TRG was determined based on consensus between the two pathologists. In cases of disagreement, a third senior pathologist was consulted, and a consensus decision was reached.

### 2.4 Data collection

Baseline and post–neoadjuvant treatment data were collected for all patients, including demographic characteristics, tumor-related variables, and nutritional indicators.

Baseline characteristics included sex, age, height, weight, smoking history, alcohol consumption history, and history of hypertension. Tumor-related variables included tumor location, tumor regression grade, clinical T stage and N stage before neoadjuvant therapy, and serum tumor markers [carcinoembryonic antigen (CEA) and carbohydrate antigen 19–9 (CA19-9)]. Nutritional indicators included hemoglobin concentration, total protein concentration, albumin concentration, prealbumin concentration, globulin concentration, albumin-toglobulin ratio (AGR) and the Geriatric Nutritional Risk Index (GNRI).

The GNRI formula is as follows:


GNRI=[1.489×albumin(g/L)]+[41.7

×(weight/idealweight)]

Ideal weight was calculated from the Lorentz equations (WLo) as follows:


Formen:idealweight=height-100-[(height-150)/4)]

For⁢women:ideal⁢weight=height-100-

[(height-150)/2.5)]

In addition, dynamic changes in these variables before and after neoadjuvant treatment were calculated and analyzed (Δx = post-treatment value - pre-treatment value).

### 2.5 Data preprocessing

Among the 255 enrolled patients, 60 achieved complete response, while 195 did not. The proportion of missing data for all variables was less than 30%. Among all the variables, only CEA and CA199 had a small number of missing values: CEA was missing in 3 cases (1.2%), and CA199 was missing in 2 cases (0.8%). Given the very low proportion of missing data in this study, missing values for variables with a normal distribution were imputed using the mean, while those for variables with a non-normal distribution were imputed using the median.

Due to the wide range and extreme values observed in tumor marker levels, logarithmic transformation was applied to CEA and CA19-9 to reduce the influence of outliers and to compress highly dispersed positive values into a narrower dynamic range.

### 2.6 Variable selection

In the training cohort, univariate analyses were first performed for all candidate variables, and variables with a *p* < 0.05 were preliminarily retained. These variables were then entered into a multivariate logistic regression analysis, and variables that remained statistically significant (*p* < 0.05) were selected for model construction.

### 2.7 Model construction and performance evaluation

Selected variables were used to construct a logistic regression model. The area under the receiver operating characteristic curve (AUC) was used as the primary metric to evaluate the discriminative ability of the model. Model accuracy, sensitivity, specificity, negative predictive value, and positive predictive value were also calculated to comprehensively assess model performance.

Calibration curves were generated to evaluate the agreement between predicted probabilities and observed outcomes. Decision curve analysis was performed to assess the clinical utility of the model. To enhance clinical applicability, a nomogram was developed based on the final model.

### 2.8 Statistical analysis

Statistical analyses were performed using SPSS version 25.0 and R version 4.5.0. Continuous variables with a normal distribution and homogeneous variances were compared using the independent samples *t*-test and are presented as mean ± standard deviation (mean ± SD). Continuous variables with non-normal distributions or heterogeneous variances were analyzed using the rank-sum test and are presented as median with interquartile range [M (P25, P75)]. Categorical variables were compared using the chi-square test and are presented as frequencies and percentages. A two-sided *p* < 0.05 was considered statistically significant.

## 3 Results

### 3.1 Comparison of baseline clinical characteristics

As shown in Table 1, no statistically significant differences were observed in baseline clinical characteristics between the training cohort and the validation cohort among the 255 patients with colorectal cancer (all *P* > 0.05), indicating that the cohort allocation was random and comparable.

**TABLE 1:** Comparison of clinical characteristics between training and validation cohorts in locally advanced MSS/pMMR colorectal cancer patients with neoadjuvant immunotherapy.

Variables	Total (*n* = 255)	test (*n* = 77)	train (*n* = 178)	Statistic	*P*
Pre-weight (kg)	60.94 ± 9.90	60.43 ± 10.16	61.15 ± 9.80	*t* = −0.53	0.594
Pre-TP (g/L)	70.09 ± 5.38	69.72 ± 5.40	70.25 ± 5.38	*t* = −0.72	0.472
Pre-ALB (g/L)	40.37 ± 3.73	39.99 ± 3.49	40.53 ± 3.83	*t* = −1.07	0.288
Pre-PA (mg/L)	228.01 ± 72.42	223.58 ± 76.95	229.93 ± 70.51	*t* = −0.64	0.522
Pre-GNRI	103.51 ± 8.80	103.23 ± 9.06	103.63 ± 8.71	*t* = −0.34	0.735
Post-weight (kg)	60.29 ± 9.63	59.82 ± 10.28	60.49 ± 9.36	*t* = −0.51	0.612
Post-PA (mg/L)	236.99 ± 76.26	239.35 ± 79.36	235.97 ± 75.08	*t* = 0.32	0.746
Post-AGR	1.30 ± 0.28	1.30 ± 0.30	1.30 ± 0.27	*t* = 0.10	0.923
ΔTP	0.55 ± 7.81	1.58 ± 7.90	0.11 ± 7.75	*t* = 1.39	0.166
Age (year)	57.00 (49.00, 66.00)	57.00 (48.00, 65.00)	57.00 (49.00, 67.25)	*Z* = −0.25	0.806
Pre-HGB (g/L)	119.60 (102.00, 133.20)	118.00 (108.00, 131.80)	120.15 (101.00, 133.20)	*Z* = −0.04	0.966
Pre-Glo (g/L)	29.30 (26.65, 32.70)	29.60 (26.30, 33.20)	29.15 (27.00, 32.25)	*Z* = −0.29	0.769
Pre-LogCEA	0.80 (0.49, 1.27)	0.83 (0.52, 1.28)	0.80 (0.48, 1.24)	*Z* = −0.51	0.608
Pre-LogCA199	0.91 (0.56, 1.45)	1.08 (0.68, 1.48)	0.89 (0.51, 1.42)	*Z* = −1.40	0.163
Pre-AGR	1.37 (1.21, 1.55)	1.34 (1.20, 1.50)	1.38 (1.22, 1.56)	*Z* = −1.14	0.253
Post-HGB (g/L)	110.40 (94.15, 126.10)	110.00 (94.00, 127.00)	110.40 (94.35, 126.00)	*Z* = −0.08	0.934
Post-TP (g/L)	71.10 (66.80, 74.85)	72.10 (67.70, 75.00)	70.70 (66.40, 74.75)	*Z* = −1.06	0.289
Post-ALB (g/L)	40.00 (36.55, 42.60)	40.70 (37.00, 43.00)	39.50 (36.35, 42.55)	*Z* = −0.91	0.362
Post-Glo (g/L)	30.90 (27.15, 34.75)	30.90 (27.00, 36.40)	30.85 (27.30, 34.27)	*Z* = −0.30	0.766
Post-LogCEA	0.54 (0.36, 0.76)	0.59 (0.42, 0.77)	0.53 (0.35, 0.74)	*Z* = −1.49	0.135
Post-LogCA199	0.92 (0.52, 1.26)	0.98 (0.72, 1.37)	0.86 (0.47, 1.18)	*Z* = −1.94	0.053
Post-GNRI	103.25 (94.91, 108.58)	103.64 (93.23, 111.19)	102.86 (95.82, 107.88)	*Z* = −0.86	0.392
ΔHGB	−5.30 (−17.30, 4.00)	−4.10 (−18.20, 5.00)	−6.35 (−16.97, 3.10)	*Z* = −0.15	0.883
ΔALB	−1.20 (−4.20, 1.75)	−1.00 (−2.90, 3.60)	−1.40 (−4.30, 1.48)	*Z* = −1.63	0.104
ΔGlo	1.30 (−1.75, 5.00)	1.30 (−1.50, 5.80)	1.25 (−1.87, 4.90)	*Z* = −0.55	0.581
ΔPA	7.90 (−38.15, 56.50)	7.90 (−42.00, 74.90)	7.05 (−31.35, 49.50)	*Z* = −0.67	0.501
ΔCEA	−1.58 (−12.08, 0.55)	−1.95 (−11.63, 0.41)	−1.39 (−12.67, 0.63)	*Z* = −0.43	0.667
ΔLogCEA	−0.17 (−0.59, 0.04)	−0.18 (−0.60, 0.04)	−0.17 (−0.58, 0.04)	*Z* = −0.32	0.753
ΔCA199	0.00 (−10.23, 2.03)	0.00 (−10.67, 1.94)	0.00 (−10.07, 2.04)	*Z* = −0.17	0.868
ΔLogCA199	0.00 (−0.32, 0.13)	0.00 (−0.29, 0.11)	0.00 (−0.34, 0.15)	Z = −0.15	0.884
ΔGNRI	−1.92 (−7.09, 2.98)	−0.86 (−5.62, 4.27)	−2.42 (−7.26, 2.43)	*Z* = −1.35	0.176
ΔAGR	−0.08 (−0.25, 0.07)	−0.06 (−0.29, 0.13)	−0.09 (−0.23, 0.04)	*Z* = −0.91	0.361
Sex, n(%)				χ^2^ = 1.44	0.231
Male	163 (63.92)	45 (58.44)	118 (66.29)		
Female	92 (36.08)	32 (41.56)	60 (33.71)		
Smoking, n(%)				χ^2^ = 0.00	0.997
No	162 (63.53)	48 (62.34)	111 (62.36)		
Yes	93 (36.47)	29 (37.66)	67 (37.64)		
Drinking, n(%)				χ^2^ = 3.15	0.076
No	179 (70.20)	60 (77.92)	119 (66.85)		
Yes	76 (29.80)	17 (22.08)	59 (33.15)		
Hypertension, n(%)				χ^2^ = 0.76	0.382
No	207 (81.18)	60 (77.92)	147 (82.58)		
Yes	48 (18.82)	17 (22.08)	31 (17.42)		
T stage, n(%)				χ^2^ = 0.79	0.373
T3	153 (60.00)	43 (55.84)	110 (61.80)		
T4	102 (40.00)	34 (44.16)	68 (38.20)		
N stage, n(%)				χ^2^ = 4.68	0.096
N0	8 (3.14)	5 (6.49)	3 (1.69)		
N1	88 (34.51)	23 (29.87)	65 (36.52)		
N2	159 (62.35)	49 (63.64)	110 (61.80)		
Location, n(%)				χ^2^ = 1.40	0.237
Right	193 (75.69)	62 (80.52)	131 (73.60)		
Left	62 (24.31)	15 (19.48)	47 (26.40)		

Pre-weight, pre-treatment weight; Pre-HGB, hemoglobin concentration; Pre-TP, pre-treatment total protein concentration; Pre-ALB, pre-treatment albumin concentration; Pre-PA, pre-treatment prealbumin concentration; Pre-Glo, pre-treatment globulin concentration; Pre-AGR, pre-treatment albumin-toglobulin ratio; Pre-GNRI, pre-treatment Geriatric Nutritional Risk Index; Pre-LogCEA, pre-treatment LogCEA; Post-weight, post-treatment weight; Post-HGB, post-treatment hemoglobin concentration; Post-TP, post-treatment total protein concentration; Post-ALB, post-treatment albumin concentration; Post-PA, post-treatment prealbumin concentration; Post-Glo, post-treatment globulin concentration; Post-AGR, post-treatment albumin-toglobulin ratio; Post-GNRI, post-treatment Geriatric Nutritional Risk Index; Post-LogCEA, post-treatment LogCEA; Δx, post-treatment value—pre-treatment value.

### 3.2 Univariate analysis

The results of univariate analysis in the training cohort are presented in Table 2. Patients in the pCR group were significantly younger than those in the non-pCR group. Before neoadjuvant treatment, there were no significant differences between the pCR and non-pCR groups in hemoglobin concentration, total protein concentration, albumin concentration, prealbumin concentration, globulin concentration, AGR, log-transformed CEA (LogCEA), log-transformed CA19-9 (LogCA19-9), or GNRI.

**TABLE 2:** Comparison of clinical characteristics between pCR group and non-pCR group in the training cohort.

Variables	Total (*n* = 178)	pCR group (*n* = 132)	Non-pCR Group (*n* = 46)	Statistic	*P*
Age (year)	56.55 ± 12.31	58.56 ± 11.43	50.78 ± 13.03	*t* = 3.83	**< 0.001**
Pre-weight (kg)	61.15 ± 9.80	61.38 ± 9.62	60.51 ± 10.38	*t* = 0.52	0.604
Pre-TP (g/L)	70.25 ± 5.38	70.18 ± 5.31	70.46 ± 5.64	*t* = −0.29	0.768
Pre-ALB (g/L)	40.53 ± 3.83	40.65 ± 3.85	40.18 ± 3.80	*t* = 0.72	0.472
Pre-PA (mg/L)	229.93 ± 70.51	229.97 ± 70.95	229.82 ± 69.99	*t* = 0.01	0.990
AGR	1.39 ± 0.27	1.41 ± 0.27	1.35 ± 0.27	*t* = 1.39	0.166
Post-weight (kg/L)	60.49 ± 9.36	60.08 ± 9.29	61.67 ± 9.56	*t* = −1.00	0.321
Post-HGB (g/L)	109.41 ± 20.21	107.37 ± 20.72	115.28 ± 17.58	*t* = −2.31	**0.022**
Post-PA (mg/L)	235.97 ± 75.08	225.60 ± 74.87	265.72 ± 68.09	*t* = −3.20	**0.002**
Post-AGR	1.30 ± 0.27	1.30 ± 0.28	1.29 ± 0.21	*t* = 0.46	0.648
ΔTP	0.11 ± 7.75	−0.62 ± 7.96	2.20 ± 6.76	*t* = −2.14	**0.033**
Pre-HGB (g/L)	120.15 (101.00, 133.20)	120.00 (98.45, 132.00)	122.70 (104.05, 140.50)	*Z* = −0.63	0.529
Pre-Glo (g/L)	29.15 (27.00, 32.25)	29.05 (27.08, 31.70)	29.95 (26.48, 33.10)	*Z* = −1.03	0.305
Pre-LogCEA	0.80 (0.48, 1.24)	0.77 (0.49, 1.18)	0.91 (0.43, 1.36)	*Z* = −0.57	0.569
Pre-LogCA199	0.89 (0.51, 1.42)	0.90 (0.50, 1.38)	0.81 (0.53, 1.56)	*Z* = −0.44	0.658
Pre-GNRI	104.45 (97.89, 109.31)	105.87 (98.47, 109.57)	100.71 (95.93, 107.85)	*Z* = −1.90	0.057
Post-TP (g/L)	70.70 (66.40, 74.75)	70.25 (65.90, 74.23)	72.15 (68.88, 76.65)	*Z* = −2.53	**0.011**
Post-ALB (g/L)	39.50 (36.35, 42.55)	38.90 (35.98, 42.30)	41.05 (37.75, 43.10)	*Z* = −2.30	**0.021**
Post-Glo (g/L)	30.85 (27.30, 34.27)	30.30 (26.87, 34.35)	32.00 (28.52, 34.03)	*Z* = −1.52	0.128
Post-LogCEA	0.53 (0.35, 0.74)	0.56 (0.37, 0.80)	0.47 (0.28, 0.54)	*Z* = −3.30	**< 0.001**
Post-LogCA199	0.86 (0.47, 1.18)	0.85 (0.46, 1.26)	0.90 (0.52, 1.09)	*Z* = −0.14	0.890
Post-GNRI	102.86 (95.82, 107.88)	102.41 (94.56, 107.46)	103.51 (99.38, 108.89)	*Z* = −1.47	0.141
ΔHGB	−6.35 (−16.97, 3.10)	−8.70 (−17.20, 3.00)	−2.00 (−15.73, 8.30)	*Z* = −1.70	0.088
ΔALB	−1.40 (−4.30, 1.48)	−2.00 (−4.82, 0.60)	0.45 (−3.60, 2.82)	*Z* = −2.87	**0.004**
ΔGlo	1.25 (−1.87, 4.90)	0.75 (−1.55, 4.17)	3.10 (−2.38, 5.40)	*Z* = −1.10	0.273
ΔPA	7.05 (−31.35, 49.50)	0.00 (−47.20, 33.55)	26.05 (−18.67, 75.58)	*Z* = −3.10	**0.002**
ΔCEA	−1.39 (−12.67, 0.63)	−0.91 (−7.88, 1.12)	−5.03 (−20.00, 0.11)	*Z* = −2.65	**0.008**
ΔLogCEA	−0.17 (−0.58, 0.04)	−0.10 (−0.48, 0.04)	−0.39 (−0.91, 0.03)	*Z* = −2.23	**0.026**
ΔCA199	0.00 (−10.07, 2.04)	0.00 (−6.76, 2.13)	−0.00 (−22.31, 1.49)	*Z* = −1.06	0.290
ΔLogCA199	0.00 (−0.34, 0.15)	0.00 (−0.33, 0.15)	−0.00 (−0.51, 0.12)	*Z* = −0.89	0.375
ΔGNRI	−2.42 (−7.26, 2.43)	−4.15 (−9.08, 0.36)	2.39 (−2.68, 4.91)	*Z* = −3.98	**< 0.001**
ΔAGR	−0.09 (−0.23, 0.04)	−0.09 (−0.23, 0.03)	−0.08 (−0.20, 0.03)	*Z* = −0.41	0.679
Sex, n(%)				χ^2^ = 0.82	0.364
Male	118 (66.29)	85 (64.39)	33 (71.74)		
Female	60 (33.71)	47 (35.61)	13 (28.26)		
Smoking, n(%)				χ^2^ = 0.06	0.809
No	111 (62.36)	83 (62.88)	28 (60.87)		
Yes	67 (37.64)	49 (37.12)	18 (39.13)		
Drinking, n(%)				χ^2^ = 0.67	0.414
No	119 (66.85)	86 (65.15)	33 (71.74)		
Yes	59 (33.15)	46 (34.85)	13 (28.26)		
Hypertension, n(%)				χ^2^ = 3.28	0.070
No	147 (82.58)	105 (79.55)	42 (91.30)		
Yes	31 (17.42)	27 (20.45)	4 (8.70)		
T stage, n(%)				χ^2^ = 2.43	0.119
T3	110 (61.80)	86 (65.15)	24 (52.17)		
T4	68 (38.20)	46 (34.85)	22 (47.83)		
N stage, n(%)				−-	0.477
N0	3 (1.69)	2 (1.52)	1 (2.17)		
N1	65 (36.52)	51 (38.64)	14 (30.43)		
N2	110 (61.80)	79 (59.85)	31 (67.39)		
Location, n(%)				χ^2^ = 1.23	0.268
Right	131 (73.60)	100 (75.76)	31 (67.39)		
Left	47 (26.40)	32 (24.24)	15 (32.61)		

Pre-weight, pre-treatment weight; Pre-HGB, hemoglobin concentration; Pre-TP, pre-treatment total protein concentration; Pre-ALB, pre-treatment albumin concentration; Pre-PA, pre-treatment prealbumin concentration; Pre-Glo, pre-treatment globulin concentration; Pre-AGR, pre-treatment albumin-toglobulin ratio; Pre-GNRI, pre-treatment Geriatric Nutritional Risk Index; Pre-LogCEA, pre-treatment LogCEA; Post-weight, post-treatment weight; Post-HGB, post-treatment hemoglobin concentration; Post-TP, post-treatment total protein concentration; Post-ALB, post-treatment albumin concentration; Post-PA, post-treatment prealbumin concentration; Post-Glo, post-treatment globulin concentration; Post-AGR, post-treatment albumin-toglobulin ratio; Post-GNRI, post-treatment Geriatric Nutritional Risk Index; Post-LogCEA, post-treatment LogCEA; Δx = post-treatment value—pre-treatment value. Bold values indicate *P* < 0.05, which is considered statistically significant.

After neoadjuvant treatment, hemoglobin concentration, total protein concentration, albumin concentration, and prealbumin concentration were significantly higher in the pCR group than in the non-pCR group, whereas LogCEA levels were significantly lower in the pCR group. In addition, total protein concentration, albumin concentration, prealbumin concentration, and GNRI increased after neoadjuvant treatment in the pCR group, whereas albumin concentration and GNRI decreased and prealbumin concentration showed no significant change in the non-pCR group; these differences between the two groups were statistically significant.

CEA levels decreased after neoadjuvant treatment in both the pCR and non-pCR groups, with a more pronounced decline observed in the pCR group.

### 3.3 Multivariate analysis

Clinical variables that showed statistically significant differences between the pCR and non-pCR groups in univariate analysis were entered into a multivariate logistic regression model (Table 3). The results demonstrated that age [odds ratio (OR) = 0.96, 95% confidence interval (CI): 0.93–0.99], post–neoadjuvant treatment LogCEA (OR = 0.19, 95% CI: 0.05–0.70), and GNRI change after neoadjuvant treatment (ΔGNRI) (OR = 1.07, 95% CI: 1.02–1.12) were independent predictors of achieving pCR in patients with locally advanced colorectal cancer treated with neoadjuvant immunotherapy.

**TABLE 3:** Multivariate analysis of pCR after neoadjuvant immunotherapy for locally advanced MSS/pMMR colorectal cancer patients in the training cohort.

Variables	OR (95%CI)	*P*
Age (year)	0.96 (0.93 ∼ 0.99)	**0.020**
Post-LogCEA	0.19 (0.05 ∼ 0.70)	**0.013**
ΔGNRI	1.07 (1.02 ∼ 1.12)	**0.006**

OR, odds ratio; CI, confidence interval; Post-LogCEA, post–neoadjuvant treatment LogCEA. Bold values indicate *P* < 0.05, which is considered statistically significant.

### 3.4 Model construction and predictive performance

**FIGURE 2:**
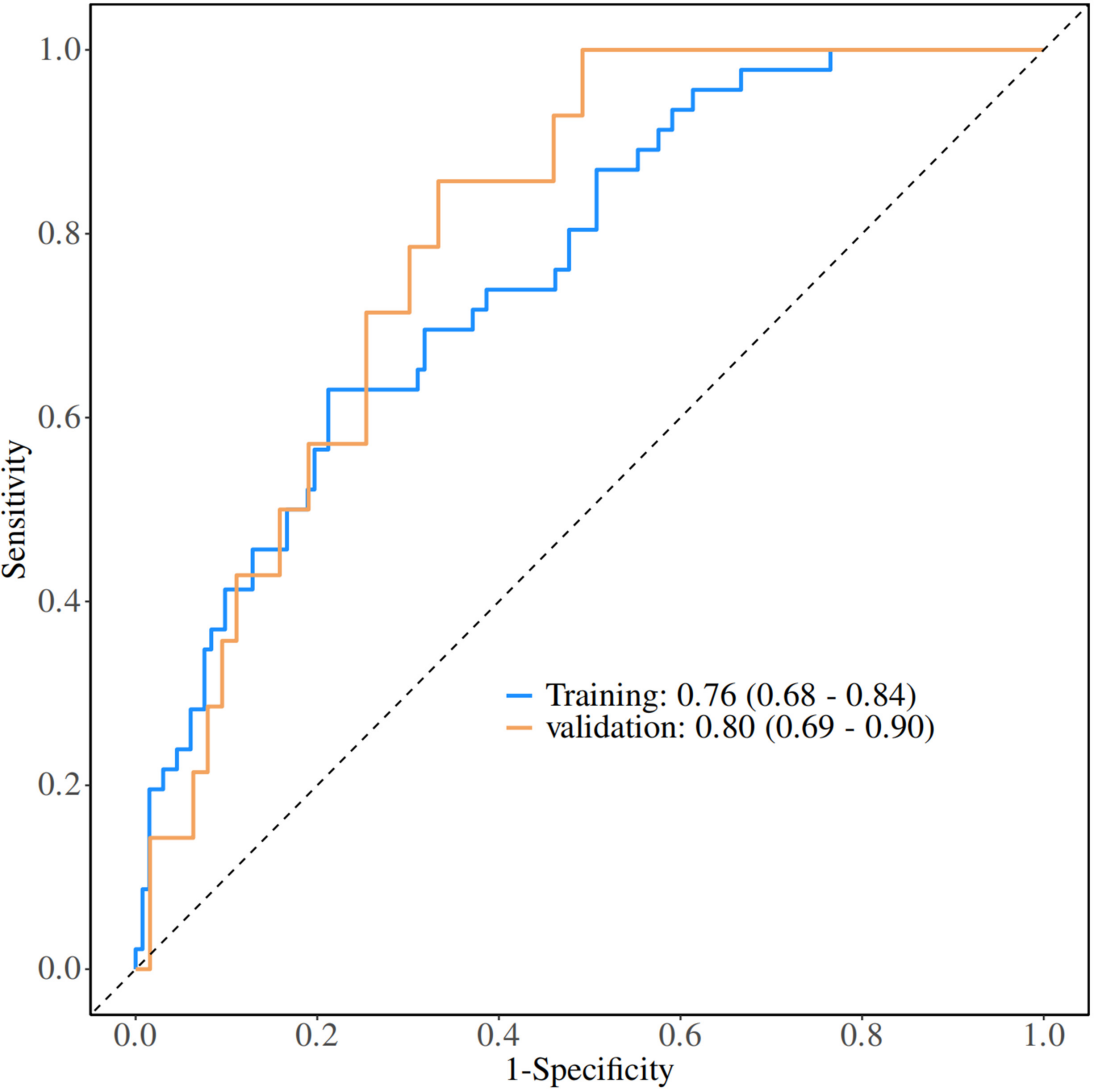
The AUC for the LR model in the training and validation cohorts.

Independent predictors identified in the multivariate analysis were used to construct a logistic regression (LR) model. The model achieved an area under the receiver operating characteristic curve (AUC) of 0.76 in the training cohort and 0.80 in the validation cohort (Figure 2).

In the training cohort, the accuracy, sensitivity, specificity, positive predictive value (PPV), and negative predictive value (NPV) were 0.75 (95% CI: 0.68–0.81), 0.63 (95% CI: 0.49–0.77), 0.79 (95% CI: 0.72–0.86), 0.51 (95% CI: 0.38–0.64), and 0.86 (95% CI: 0.80–0.92), respectively (Table 4).

**TABLE 4:** Performance of logistic regression model in the training and validation cohorts.

Data	Accuracy (95%CI)	Sensitivity (95%CI)	Specificity (95%CI)	PPV (95%CI)	NPV (95%CI)
Train	0.75 (0.68–0.81)	0.63 (0.49–0.77)	0.79 (0.72–0.86)	0.51 (0.38 −0.64)	0.86 (0.80 −0.92)
Test	0.73 (0.61–0.82)	0.64 (0.39–0.89)	0.75 (0.64–0.85)	0.36 (0.17 −0.55)	0.90 (0.82 −0.98)

PPV, positive predictive value; NPV, negative predictive value.

In the validation cohort, the accuracy, sensitivity, specificity, PPV, and NPV were 0.73 (95% CI: 0.61–0.82), 0.64 (95% CI: 0.39–0.89), 0.75 (95% CI: 0.64–0.85), 0.36 (95% CI: 0.17–0.55), and 0.90 (95% CI: 0.82–0.98), respectively (Table 4).

**FIGURE 3:**
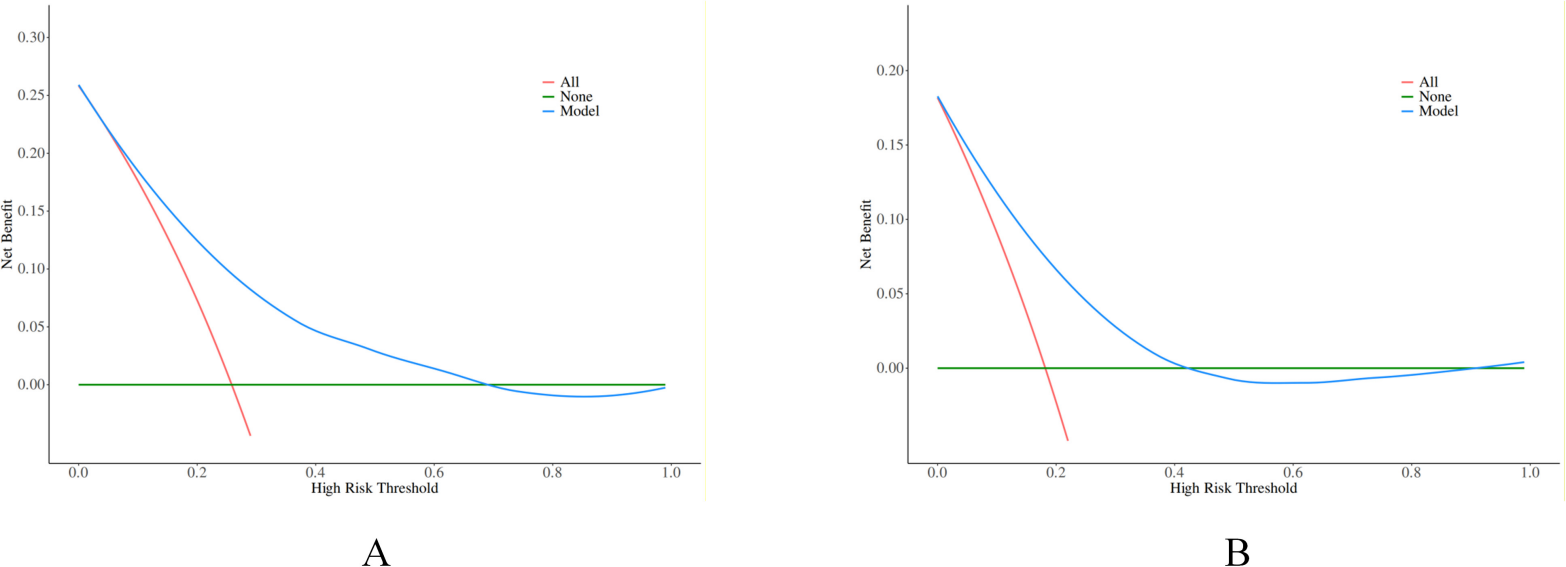
The DCA for the LR model in the training **(A)** and validation **(B)** cohorts.

**FIGURE 4:**
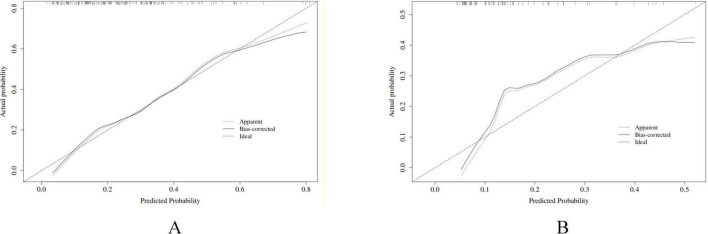
The Calibration curve analysis for the LR model in the training **(A)** and validation **(B)** cohorts.

Decision curve analysis (DCA) demonstrated that the net benefit of the constructed model consistently exceeded those of the “treat-all” and “treat-none” strategies across a wide range of threshold probabilities, indicating favorable clinical utility (Figure 3). Calibration curve analysis showed that the bias-corrected prediction curve closely aligned with the ideal reference line, suggesting favorable agreement between predicted probabilities and observed outcomes and good calibration performance (Figure 4).

### 3.5 Nomogram of the logistic regression model

**FIGURE 5:**
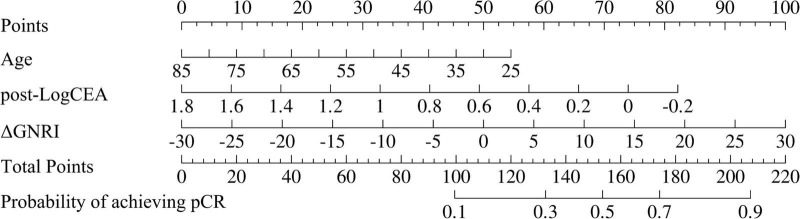
Nomogram constructed based on the LR model for prediction of pCR in locally advanced MSS/pMMR colorectal cancer patients with Neoadjuvant Immunotherapy.

As shown in Figure 5, a nomogram was developed based on the final LR model to enhance the clinical interpretability of the study findings. This visual tool enables clinicians to rapidly estimate the probability of achieving pCR by integrating patient age, post–neoadjuvant treatment LogCEA, and the change in GNRI before and after neoadjuvant treatment.

## 4 Discussion

Colorectal cancer is one of the most prevalent gastrointestinal malignancies worldwide, and neoadjuvant therapy followed by surgical resection has become the standard treatment paradigm for patients with locally advanced rectal cancer. With the continuous advancement of immunotherapy, its role in the neoadjuvant setting has gained increasing attention. However, the marked heterogeneity in treatment response to neoadjuvant immunotherapy remains a major clinical challenge, and the lack of reliable predictive biomarkers has substantially hindered the implementation of personalized treatment strategies.

Systemic nutritional status is closely associated with host immune function, and the efficacy of immune checkpoint inhibitors relies on an intact and effective antitumor immune response. This biological relationship provides a strong theoretical rationale for considering nutritional indicators as potential predictors of immunotherapy efficacy (20–23). In this study, we focused on systemic nutritional indicators and their dynamic changes to preliminarily explore their value in predicting the efficacy of neoadjuvant immunotherapy in patients with locally advanced MSS/pMMR colorectal cancer and to develop a clinically applicable predictive model.

Unlike most previous studies that adopted a cross-sectional design, the present study further analyzed dynamic changes in nutritional indicators before and after treatment, which may better reflect the interaction between host systemic status and tumor response during neoadjuvant immunotherapy.

Multivariate analysis in the present study clearly identified age as an independent predictor of achieving pCR after neoadjuvant immunotherapy (OR = 0.96, 95% CI: 0.93–0.99), indicating that younger patients were more likely to achieve pCR and consequently experience more favorable prognoses. This finding is highly consistent with results from multiple previous studies on cancer immunotherapy (24–27). A potential underlying mechanism may be related to the absence of immunosenescence in younger individuals, who generally exhibit more robust immune systems characterized by higher proliferative capacity of immune cells and more active cytokine secretion. These immunological advantages may facilitate a stronger and more effective response to immune checkpoint inhibitors, thereby promoting the initiation and maintenance of antitumor immune responses (28–30).

In addition, younger patients typically possess superior tissue repair capacity and greater tolerance to treatment-related stress and injury, enabling them to complete neoadjuvant treatment regimens more effectively (31–33). This enhanced treatment compliance and physiological resilience may further create favorable conditions for achieving pCR.

CEA is a well-established serum tumor marker widely used in the clinical management of colorectal cancer, playing an important role in disease screening, assessment of tumor burden, and post-treatment surveillance (34). Numerous clinical studies have demonstrated a strong correlation between CEA levels and tumor burden as well as clinical stage in patients with colorectal cancer, and changes in CEA expression can directly reflect tumor proliferation and progression (35). In the present study, the reduction in CEA levels following neoadjuvant immunotherapy essentially indicates suppression of tumor cell proliferation and a decrease in tumor burden. The significant association between marked CEA decline and pCR further confirms the classical value of tumor markers in evaluating treatment response, even in the era of immunotherapy.

GNRI, which integrates serum albumin levels and body weight changes, is a comprehensive nutritional assessment tool that objectively reflects overall nutritional reserves and metabolic balance (36). In the present study, GNRI increased after neoadjuvant immunotherapy in patients who achieved pCR, whereas a declining trend was observed in the non-pCR group. This dynamic difference suggests that an increase or stable maintenance of GNRI essentially reflects adequate nutritional reserves, which provide sufficient energy and substrates to support immune cell activation, proliferation, and the secretion of antitumor cytokines. Such a favorable nutritional state may ensure the effective functioning of immune checkpoint inhibitors by relieving tumor-induced immune suppression and robustly activating antitumor immune responses, thereby significantly enhancing the tumor-specific cytotoxic activity of immune cells. This finding is highly consistent with the core mechanism by which immunotherapy depends on the integrity of the nutrition–immunity axis and further underscores the critical supportive role of nutritional status in determining immunotherapy efficacy (37, 38).

Multivariate logistic regression analysis identified age, post-treatment CEA levels, and dynamic changes in GNRI as independent predictors of pCR. The logistic regression model constructed based on these variables demonstrated good predictive performance in both the training and validation cohorts, with AUCs of 0.76 and 0.80, respectively. Moreover, calibration curves showed favorable agreement between predicted probabilities and observed outcomes, and decision curve analysis confirmed a significant net clinical benefit. These results indicate that the proposed model not only possesses satisfactory discriminative ability and calibration performance but also provides reference value for clinical decision-making by helping clinicians identify patients who are more likely to benefit from neoadjuvant immunotherapy, thereby avoiding unnecessary treatment-related toxicity and inefficient use of medical resources.

A nomogram derived from the established logistic regression model was developed to enhance clinical applicability through intuitive visualization. By simply inputting patient age, post–neoadjuvant treatment LogCEA, and the change in GNRI before and after treatment, clinicians can conveniently estimate the probability of achieving pCR, thereby substantially facilitating the translation of the study findings into routine clinical practice. Notably, the relative contribution of CEA was higher than that of nutritional indicators in the nomogram, suggesting that future studies should expand the scope of nutritional parameters included in the model. Incorporating additional nutrition-related factors that are more closely associated with immunotherapy response in patients with locally advanced MSS/pMMR colorectal cancer may help optimize the predictive weight of nutritional indicators, further strengthen their central role in pCR prediction, and improve the overall clinical specificity and performance of the model.

Admittedly, the model developed in this study has certain limitations for clinical application. The PPV of our model was only 0.36, meaning that only 36% of patients identified by the model as achieving pathological complete response (pCR) were confirmed to have achieved pCR by postoperative pathological examination. Relying solely on this model for subsequent clinical decisions would entail a high decision-making risk. For example, it could lead to the inappropriate selection of a watch-and-wait strategy for patients who have not actually achieved complete response (CR), thereby delaying the optimal opportunity for treatment. Notably, the NPV of our model reached 0.90, indicating a high level of agreement between the model’s prediction of not achieving pCR and the postoperative pathological findings. This suggests that when the model indicates a patient has not achieved CR, it can provide a reliable reference for clinicians to opt for direct surgical intervention rather than a watch-and-wait strategy. In summary, the model constructed in this study is not yet suitable as a basis for independent clinical decision-making. However, it can serve as a supplementary reference tool to assist clinical practice, offering valuable insights to help clinicians optimize treatment decisions.

Nevertheless, several limitations of this study should be acknowledged. First, as a single-center retrospective study, selection bias cannot be completely excluded, and the relatively limited sample size warrants external validation in multicenter, large-scale prospective studies. Second, only conventional nutritional indicators, such as hemoglobin and total protein, were included, whereas potentially relevant variables, including body composition parameters and serum inflammatory markers, were not assessed. Future studies incorporating a broader range of nutritional and inflammatory indicators may further improve model performance. Third, as this study did not conduct an in-depth investigation of intermediate markers such as tumor-infiltrating lymphocytes (TILs) and the systemic immune-inflammation index (SII), the mechanistic associations between the ΔGNRI and pCR remain preliminary and lack sufficient experimental evidence. Therefore, future systematic research is warranted to further explore the underlying mechanisms linking these indicators with pCR, thereby providing a more robust theoretical basis for optimizing the model and supporting its clinical application. Fourth, the present study did not evaluate the impact of nutritional interventions on treatment outcomes; therefore, it remains unclear whether improving nutritional status can enhance the response to neoadjuvant immunotherapy. This issue merits further investigation in future prospective trials.

## 5 Conclusion

Age, post-neoadjuvant treatment LogCEA levels and ΔGNRI are independent predictors of pCR in locally advanced MSS/pMMR colorectal cancer patients treated with neoadjuvant immunotherapy. The predictive model and nomogram developed in this study offer a valuable reference to support clinical decision-making, providing a new perspective for accurately predicting treatment response and laying a theoretical foundation for future research on nutritional interventions combined with immunotherapy.

## Data Availability

The original contributions presented in the study are included in the article/Supplementary material, further inquiries can be directed to the corresponding authors.
