# Supplementary material for: Systemic nutritional status and its dynamic changes as predictors of response to neoadjuvant immunotherapy in locally advanced MSS/pMMR colorectal cancer
Source: Front Med (Lausanne). 2026 Mar 27;13:1803929. doi: 10.3389/fmed.2026.1803929 (PMC13065721; doi:10.3389/fmed.2026.1803929)
Supplement: Supplementary file 1 [file Table_1.docx]

**TABLE S1 Comparison of treatment cycles between pCR group and non-pCR group in locally advanced MSS/pMMR colorectal cancer patients with neoadjuvant immunotherapy.**

| **Variables** | **Total (n = 255)** | **Non-pCR group (n = 195)** | **pCR group**  **(n = 60)** | **Statistic** | ***P*** |
| --- | --- | --- | --- | --- | --- |
|  |  |  |  |  |  |
| Chemotherapy alone cycles | 0.00 (0.00, 0.00) | 0.00 (0.00, 0.00) | 0.00 (0.00, 0.00) | Z=-1.10 | 0.271 |
| Chemotherapy + Immunotherapy cycles | 4.00 (4.00, 4.00) | 4.00 (4.00, 4.00) | 4.00 (4.00, 4.00) | Z=-0.73 | 0.464 |
| Total treatment cycles | 4.00 (4.00, 4.00) | 4.00 (4.00, 4.00) | 4.00 (4.00, 4.00) | Z=-1.17 | 0.242 |

Chemotherapy alone cycles: the number of cycles of chemotherapy alone (no immunotherapy), Chemotherapy + Immunotherapy cycles: the number of cycles of chemotherapy combined with immunotherapy, Total treatment cycles: The sum of the number of cycles of chemotherapy alone and the number of cycles of chemotherapy combined with immunotherapy.
