# Supplementary material for: Systemic nutritional status and its dynamic changes as predictors of response to neoadjuvant immunotherapy in locally advanced MSS/pMMR colorectal cancer
Source: Front Med (Lausanne). 2026 Mar 27;13:1803929. doi: 10.3389/fmed.2026.1803929 (PMC13065721; doi:10.3389/fmed.2026.1803929)
Supplement: Supplementary file 2 [file Table_2.docx]

**TABLE S2 Comparison of detailed treatment cycles treatment pCR group and non-pCR group in locally advanced MSS/pMMR colorectal cancer patients with neoadjuvant immunotherapy.**

| **Variables** | **Total**  **(n = 255)** | **Non-pCR group**  **(n = 195)** | **pCR group**  **(n = 60)** | **Statistic** | ***P*** |
| --- | --- | --- | --- | --- | --- |
|  |  |  |  |  |  |
| Chemotherapy alone cycles, n(%) |  |  |  | - | 0.092 |
| 0 | 238 (93.33) | 184 (94.36) | 54 (90.00) |  |  |
| 1 | 6 (2.35) | 2 (1.03) | 4 (6.67) |  |  |
| 2 | 5 (1.96) | 4 (2.05) | 1 (1.67) |  |  |
| 3 | 6 (2.35) | 5 (2.56) | 1 (1.67) |  |  |
| Chemotherapy + Immunotherapy cycles, n(%) |  |  |  | - | 0.400 |
| 1 | 16 (6.27) | 14 (7.18) | 2 (3.33) |  |  |
| 2 | 24 (9.41) | 17 (8.72) | 7 (11.67) |  |  |
| 3 | 21 (8.24) | 17 (8.72) | 4 (6.67) |  |  |
| 4 | 182 (71.37) | 139 (71.28) | 43 (71.67) |  |  |
| 5 | 6 (2.35) | 3 (1.54) | 3 (5.00) |  |  |
| 6 | 4 (1.57) | 4 (2.05) | 0 (0.00) |  |  |
| 7 | 2 (0.78) | 1 (0.51) | 1 (1.67) |  |  |
| Total Treatment cycles, n(%) |  |  |  | - | 0.126 |
| 1 | 7 (2.75) | 7 (3.59) | 0 (0.00) |  |  |
| 2 | 24 (9.41) | 17 (8.72) | 7 (11.67) |  |  |
| 3 | 18 (7.06) | 17 (8.72) | 1 (1.67) |  |  |
| 4 | 191 (74.90) | 143 (73.33) | 48 (80.00) |  |  |
| 5 | 8 (3.14) | 5 (2.56) | 3 (5.00) |  |  |
| 6 | 5 (1.96) | 5 (2.56) | 0 (0.00) |  |  |
| 7 | 2 (0.78) | 1 (0.51) | 1 (1.67) |  |  |

Chemotherapy alone cycles: the number of cycles of chemotherapy alone (no immunotherapy), Chemotherapy + Immunotherapy cycles: the number of cycles of chemotherapy combined with immunotherapy, Total treatment cycles: The sum of the number of cycles of chemotherapy alone and the number of cycles of chemotherapy combined with immunotherapy.
